# Injury Patterns and Conservative Management in Elite Handball: A Multidisciplinary Approach to Diagnosis and Rehabilitation

**DOI:** 10.3390/healthcare13111303

**Published:** 2025-05-30

**Authors:** Cătălin-Adrian Miu, Mihai Hurmuz, Luminița-Oana Miu, Daniel Ceachir, Alexandru Catalin Motofelea, Romulus-Fabian Tatu

**Affiliations:** 1 Department XV, Discipline of Orthopedics, “Victor Babeș” University of Medicine and Pharmacy Timișoara, Eftimie Murgu Square 2, 300041 Timișoara, Romania; miu.catalin@umft.ro (C.-A.M.); tatu.fabian@umft.ro (R.-F.T.); 2 Orthopedics Unit, “Dr. Victor Popescu” Emergency Military Clinical Hospital, Gheorghe Lazăr Street 7, 300080 Timișoara, Romania; d.ceachir@yahoo.com; 3 Radiology and Medical Imaging Unit, Municipal Emergency Clinical Hospital, Gheorghe Dima Street 5, 300254 Timișoara, Romania; droanamiu@yahoo.com; 4 Center for Molecular Research in Nephrology and Vascular Disease, Faculty of Medicine, “Victor Babeș” University of Medicine and Pharmacy, 300041 Timișoara, Romania; alexandru.motofelea@umft.ro

**Keywords:** handball, sports injuries, trauma management, knee joint

## Abstract

Background: Musculoskeletal injuries are frequent in handball players due to the high-impact nature of the sport. Accurate diagnosis and tailored treatment are essential for recovery. Magnetic resonance imaging (MRI) has become the gold standard for evaluating complex sports-related injuries. This case report aims to assess the role of MRI in diagnosing trauma in professional handball players and the effectiveness of individualized treatment approaches. Methods: Four male members of the “Politehnica” Timișoara first team who sustained match- or training-related injuries between January 2023 and December 2024 underwent an 1.5 T MRI. Individualized conservative protocols included rest, NSAIDs, physiotherapy, and graded kinesitherapy. Results: The first case involved a right back with a knee hematoma and a vastus lateralis tear. Conservative treatment led to recovery. The second case was a left back with peripheral neuropathy caused by hamstring avulsion at the ischial tuberosity. Conservative therapy alleviated symptoms. The third case involved a left winger with adductor muscle tears, which recovered with conservative management. The fourth case, a goalkeeper, had a type III navicular fracture misdiagnosed on radiography, correctly identified via MRI and treated conservatively. Conclusions: MRI is invaluable in diagnosing handball-related injuries, enabling accurate assessments and effective individualized treatment, resulting in early recovery.

## 1. Introduction

Handball is a team sport that places considerable physical demands on its players. It is a high-intensity game marked by rapid, high-impact movements, which significantly elevate the risk of musculoskeletal injuries, especially to the knee joint. During play, athletes engage in accelerations, directional changes, throws, jumps, and frequent physical contact, as noted in [1,2]. Sports injuries among basketball, handball, and volleyball players focused on frequency, nature, and gender/position variations. Lower limb injuries were most prevalent across all three sports. Basketball players commonly had knee and ankle injuries, handball players suffered knee injuries, and volleyball players sustained knee and foot injuries [3]. These elements contribute to a high incidence of injuries associated with the sport [4]. Handball injuries are prevalent, with knee and ankle injuries being the most common [4,5,6]. The injury incidence is higher during matches compared to training, ranging from 11.1 to 23.5 injuries per 1000 match hours [4]. Contact is the dominant injury mechanism, accounting for 51.4% of injuries [7]. Previous injuries and increased match frequency are risk factors for overuse injuries, while being female and increased training hours are associated with traumatic injuries [8]. Injury patterns differ between genders, with females reporting more serious injuries [9]. Players in wing and pivot positions experience the highest rate of contact-related injuries [7]. The injury incidence is higher in international competitions compared to national leagues [9]. These findings emphasize the need for targeted injury prevention strategies in handball. Injury surveillance data from the European Handball Federation indicate that knee injuries account for approximately 18–24% of all injuries sustained in both professional and amateur matches. In addition, ligament injuries, especially those affecting the anterior cruciate ligament (ACL), have been reported with incidences as high as 0.82 per 1000 player hours, often resulting in prolonged rehabilitation or even premature career termination.

Despite the high prevalence of knee injuries in handball, detailed studies on diagnostic and therapeutic strategies specific to this sport remain scarce compared to other high-impact sports such as soccer or basketball. Magnetic resonance imaging (MRI) has emerged as a critical diagnostic tool due to its superior ability to delineate complex musculoskeletal trauma. MRI not only provides precise visualization of injuries affecting the knee joint but also aids in identifying pathologies in adjacent structures, such as the thigh muscles, hamstrings, adductors, and the accessory navicular.

The hamstring muscles—comprising the biceps femoris, semitendinosus, and semimembranosus—originate from the ischial tuberosity and function as antagonists to the quadriceps muscle, playing a vital role in knee flexion and hip extension while enhancing the biomechanical stability of both the coxofemoral and knee joints [10]. Similarly, the adductor muscles, located in the medial compartment of the thigh, are essential for thigh adduction and pelvic stabilization, engaging intensely during pivots, accelerations, and rapid changes of direction inherent to handball.

The navicular, or tarsal scaphoid, is a key bone in the foot that supports the arch through the insertion of the tibialis posterior muscle, which acts as a strong adductor and supinator of the foot [11]. The accessory navicular, an anatomical variant, is classified into three types: type 1 is small and round, residing within the posterior tibialis tendon; type 2 is larger and connected to the navicular via a cartilage bridge; and type 3 is the most prominent, fusing to the navicular bone via a bony bridge and forming a horn-shaped structure [12].

Given the high incidence of these injuries and the critical role of early diagnosis in ensuring effective recovery, this study aims to evaluate the use of MRI in diagnosing handball-related musculoskeletal trauma and to assess the outcomes of conservative treatment approaches. We specifically focus on four case studies involving male players from the “Politehnica” Timișoara team, providing a detailed examination of injury patterns and the effectiveness of various conservative therapeutic interventions, despite the greater success of surgical therapy in this context.

## 2. Materials and Methods

### 2.1. Study Population and Case Selection

We conducted a prospective case series of all male professional handball players from the “Politehnica” Timișoara first team who sustained an acute musculoskeletal injury during an official match or team training between January 2023 and December 2024, were aged eighteen years or older at the time of injury, underwent diagnostic magnetic resonance imaging (MRI) on a 1.5 T General Electric system, and received exclusively conservative (non-surgical) treatment. All participants provided written informed consent for the use of their anonymized clinical data. Four cases were selected to illustrate both the most common injury patterns and diagnostically challenging presentations in elite handball.

### 2.2. Ethical Approval and Consent

The study protocol adhered to the Declaration of Helsinki and received approval from the Ethics Committee of “Dr. Victor Popescu” Emergency Military Clinical Hospital, Timișoara (approval code: 67; approval date: 23 May 2024). Prior to participation, each athlete signed an informed consent form covering diagnostic procedures, treatment, and publication of anonymized results.

### 2.3. MRI Protocol

All imaging was performed on a 1.5 T General Electric MRI system using dedicated surface coils appropriate for the injured region. Patients were positioned supine with the affected segment centered within the coil. Scans were acquired in the axial, sagittal, and coronal planes with slice thickness between 3 and 4 mm and an interslice gap of 0.4 to 0.6 mm; the field of view was adjusted to 16–20 cm according to anatomical region. Pulse sequences included T1-weighted images for detailed anatomy of bone, tendons, and ligaments; T2-weighted images to highlight fluid collections and soft-tissue contrast; proton-density fat-saturated sequences to delineate muscle-tendon fiber disruption and early edema; and STIR sequences for sensitive detection of bone-marrow and soft-tissue edema.

### 2.4. Image Interpretation

Two fellowship-trained musculoskeletal radiologists independently reviewed each MRI study, blinded to the clinical findings. Lesions were classified by anatomical location, graded for severity (I–III for muscle strains), and evaluated for the presence and extent of associated hematoma or edema. Discrepancies in interpretation were resolved by consensus, ensuring uniform characterization across all four cases.

## 3. Results

### 3.1. Case 1: Vastus Lateralis Tear with Prepatellar Hematoma

**Figure 1:**
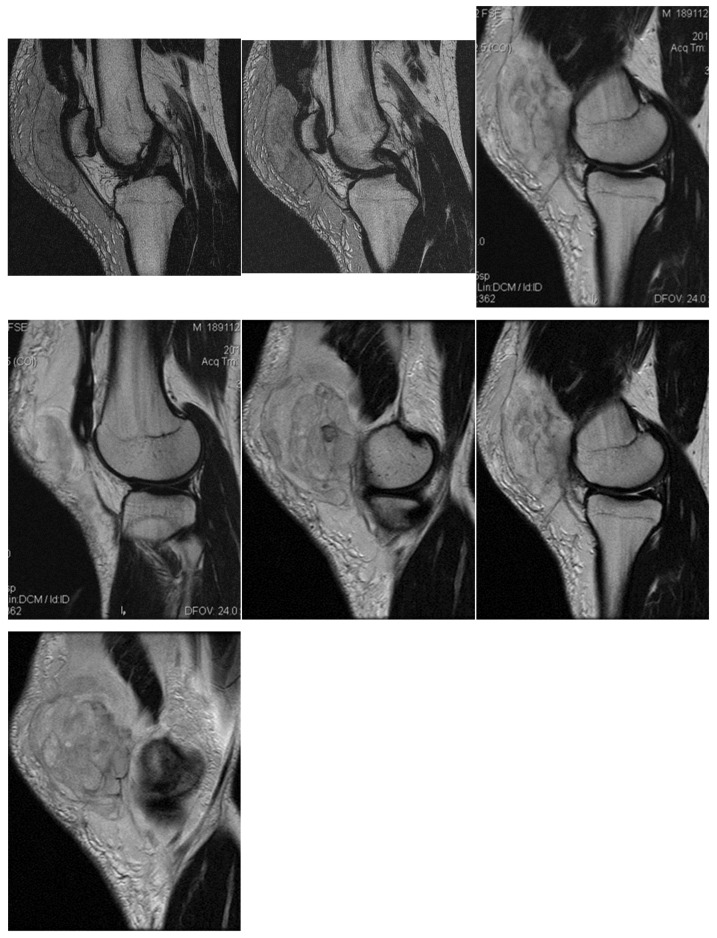
Sagittal T2 acquisition.

**Figure 2:**
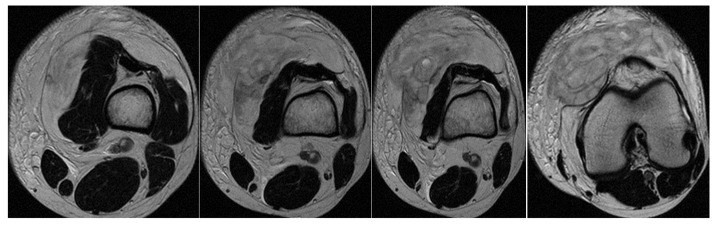
Knee MRI: axial T2 acquisition.

**Figure 3:**
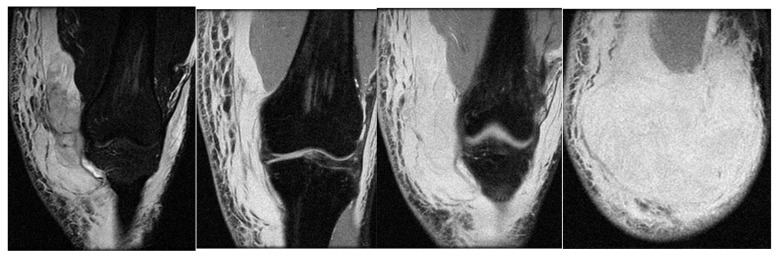
Knee MRI: coronal stir acquisition.

Right back, 22 years old, national-team player from a rural area, professional first-league athlete, 198 cm, 110 kg. In the initial case, the right back sustained injuries to the knee and the distal portion of the left thigh during a training session, attributed to a lateral concussion and the “opening” of the internal compartment. The clinical examination revealed pain, swelling, edema, a bubble knee, relative functional impairment, and restricted active and passive movement of the left knee. A sprain of the left knee was suspected, with potential damage to the meniscus, cruciate ligaments, and collateral ligaments. Although the MRI did not indicate any damage to the intra-articular structures, it did reveal a substantial prepatellar extraarticular hematoma, resulting from a muscle tear in the vastus lateralis muscle, along with significant edema of the soft tissue. The extraarticular edema is evident in the non-homogeneous Ts and STIR hyperintense, accompanied by T1 hypo- and hyperintense. Post-traumatic edema of the adjacent soft tissue is depicted in STIR as an altered diffused hyperintense (Figure 1, Figure 2 and Figure 3).

Treatment consisted of myorelaxant medication, cryotherapy, and local applications of heparin gel in the first stage, followed by physical and kinetotherapy to prevent the hematoma from becoming chronic and infected and regain knee mobility and return to full training after 6 weeks.

### 3.2. Case 2: Proximal Hamstring Avulsion with Sciatic Neuritis

**Figure 4:**
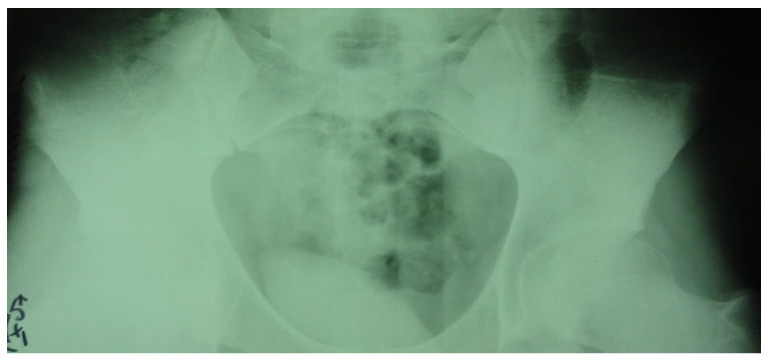
Incorrect pelvic X-ray.

In the second case, the patient, a left back, experienced pain in the inguinal region and left thigh while running during a national team training session. This 23-year-old national team member has six years of experience as a first-league professional in Romania, standing 202 cm tall and weighing 105 kg. Pelvic radiography was incorrectly performed (incorrectly) and the patient was diagnosed with left thigh muscle damage. (Figure 4).

Three years post-trauma, the player develops symptoms of peripheral neuropathy with sensory and motor symptoms in the external popliteal sciatic nerve. Lumbar degenerative disc disorder or piriformis syndrome was suspected, and lumbar and pelvic MRI was performed.

**Figure 5:**
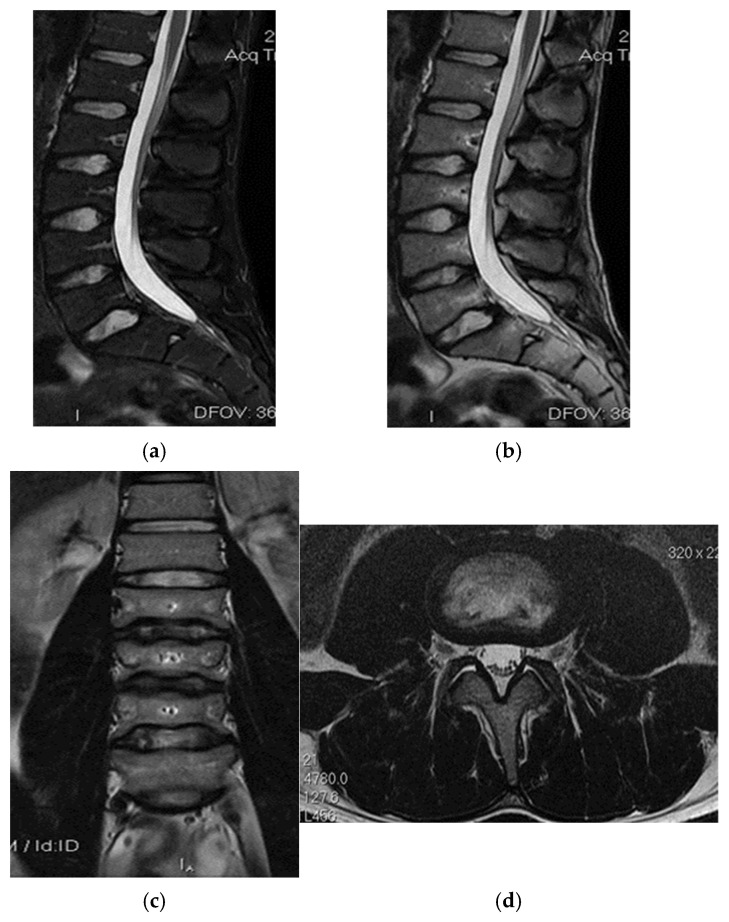
(**a**) Spinal MRI: sagittal T2 fat sat acquisition, (**b**) spinal MRI: sagittal T2 acquisition, (**c**) spinal MRI: coronal T2 acquisition, (**d**) spinal MRI: axial T2 acquisition.

**Figure 6:**
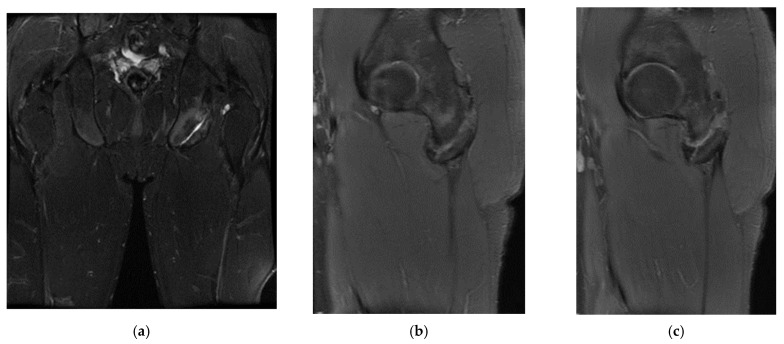

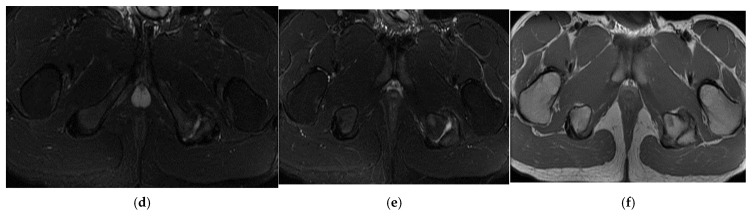
(**a**) Pelvic MRI: coronal stir acquisition, (**b**) pelvic MRI: sagittal PD fat sat acquisition, (**c**) pelvic MRI: sagittal PD fat sat acquisition, (**d**) axial stir acquisition, (**e**) axial stir acquisition, (**f**) axial T1 acquisition.

**Figure 7:**
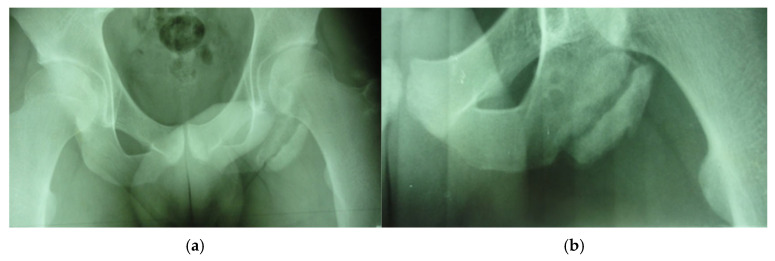
(**a**) Pelvic X-ray—ischial tuberosity, (**b**) ischial tuberosity X-ray—detail.

The spinal MRI revealed no evidence of degenerative lumbar disc pathology or radiculopathy (Figure 5). Magnetic resonance imaging (MRI) and radiographic examination of the pelvis revealed an old avulsion injury with a detached bone fragment at the insertion site on the left ischial tuberosity, involving the semitendinosus, semimembranosus, and biceps femoris muscles (collectively referred to as the ischium-calf muscles). This condition has led to the development of pseudoarthrosis and bone edema at the level of the ischial tuberosity. The sciatic nerve is positioned tangentially to the inflammatory response. Despite the detachment, the bone fragment remains vascularized due to the maintained insertion of the ischial calf muscles, preventing it from becoming a bone splinter (Figure 6 and Figure 7).

The pseudoarthritis secondary to the old ischial tuberosity avulsion can be visualized as follows:

- An old detached bone fragment with signal in the T1 and T2 ponderations, similar to the rest of the existing bone structures, but showing focal alterations of the PD fat sac and STIR hypersignal, indicating bone edema.
- Fluid accumulation in T2 hypersignal, T1 hyposignal, between the torn bone fragment and the remaining tuberosity
- The altered PD fat sac hypersignal in the sciatic nerve, adjacent to the inflammatory process, indicates changes suggestive of focal neuritis.

Surgery might be applied either for the surgical treatment of pseudoarthritis or the excision of the detached fragment with reinsertion of the ischial–calf muscles, both treatment variants were refused by the patients due to long-term postoperative recuperation, as the player was training for international competitions.

Treatment eventually consisted of rest and NSAIDs; the inflammatory process decreased in size and, consequently, contact with the sciatic nerve. The radicular phenomena progressively decreased until complete remission was achieved and returned to play after 3 weeks.

### 3.3. Case 3: Adductor Complex Strain

**Figure 8:**
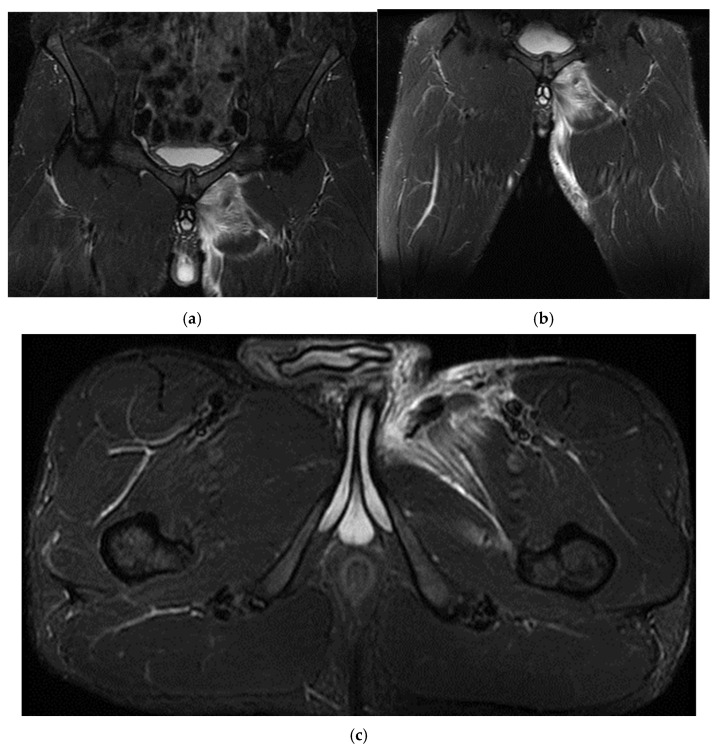
(**a**) Thigh MRI: coronal stir acquisition, (**b**) thigh MRI: coronal stir acquisition, (**c**) thigh MRI: axial stir acquisition.

In the third case, a left winger reported experiencing sharp pain in the inner left thigh, which severely impaired both active and passive movements during sprints. The left winger, aged 32, has 14 years of experience as a first-league professional in Romania and is a national team member from an urban area, standing 189 cm tall and weighing 90 kg. MRI confirmed the diagnosis of tears and disinsertion of the short adductor tendon and I- and II-degree tears of the obturator externus, pectineus, and adductor longus muscles. STIR sequences show the disinsertion of the short adductor tendon with a gap between the tendon and pubis, with the presence of significant hyperintense alterations, which were also present in the obturator externus, pectineus, and adductor brevis muscles, representing various degrees of fiber tears associated with blood clots (Figure 8).

As the reinsertion of the adductor brevis muscle involved an unacceptable period of incapacitation, the adductor magnus, the strongest muscle in the adductor group, being unimpaired, conservative treatment was applied, the player started a physical therapy program focusing on healing first- and second-degree muscle damage, respectively, the hypertonicity and hypertrophy of the other adductor muscles, mainly of the adductor magnus, the strongest muscle in the group. He returned to play after 6 weeks.

### 3.4. Case 4: Type III Accessory Navicular Impingement

A 26-year-old goalkeeper with a navicular injury, boasting six years of experience as a first-league professional in Romania, hails from an urban area and stands at 192 cm, weighing 95 kg. The latest case involves this goalkeeper, who, after sustaining a direct contact trauma during a match, reports experiencing pain and swelling in the dorsal part of his right forefoot. Forefoot X-ray diagnoses an improperly healed old right navicular fracture. As there was no trauma of the right foot, an MRI was performed, confirming the diagnosis of type III accessory navicular bone with impingement on the posterior tibial tendon.

**Figure 9:**
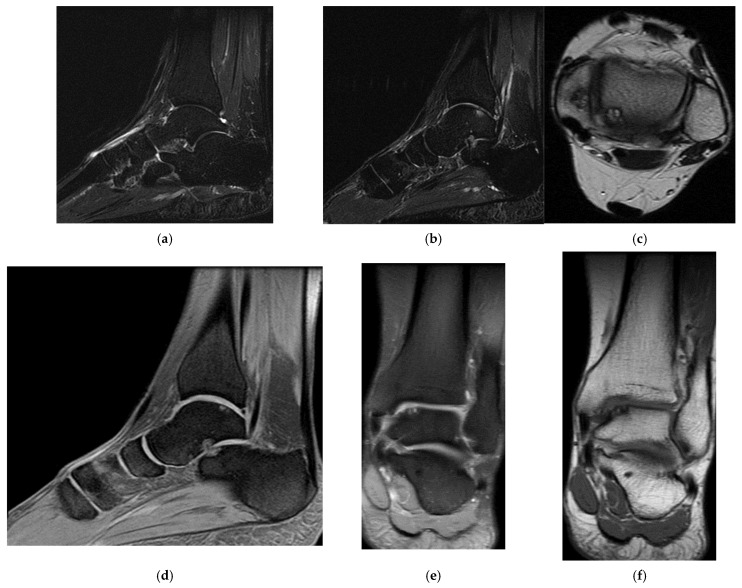
(**a**) Ankle MRI: sagittal stir acquisition, (**b**) ankle MRI: sagittal stir acquisition, (**c**) ankle MRI: axial T2 acquisition, (**d**) ankle MRI: sagittal T2 fat sat acquisition, (**e**) ankle MRI: coronal PD fat sat acquisition, (**f**) ankle MRI: coronal T1.

**Figure 10:**
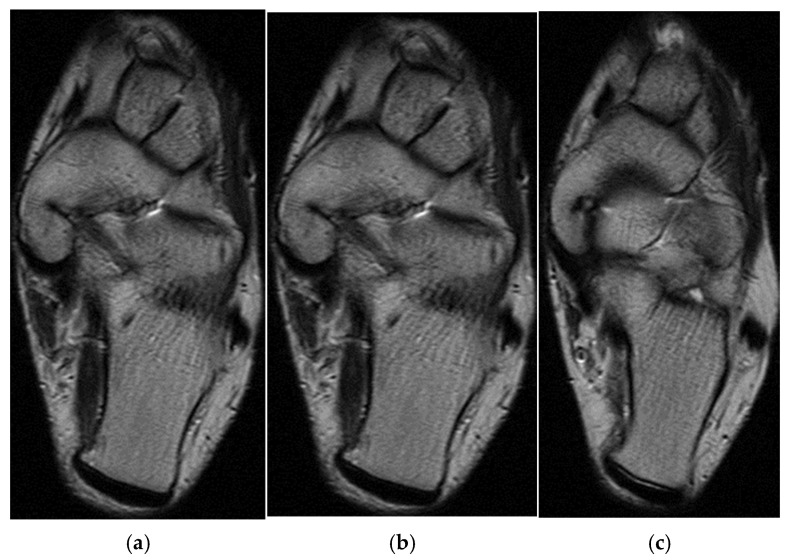
(**a**) Ankle MRI: axial T2 acquisition, (**b**) ankle MRI: axial T2 acquisition, (**c**) ankle MRI axial T2 acquisition.

**Figure 11:**
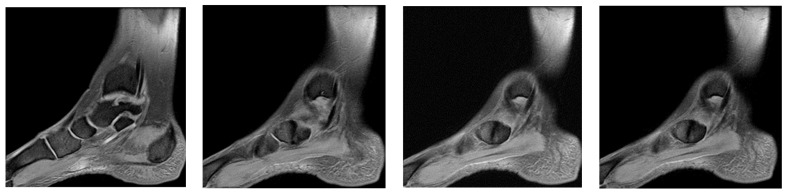
Ankle MRI: sagittal T2 fat sat acquisition.

Ankle MRI showed a well-delimited oval formation, at the posterior part of the navicular, with similar signals to the latter in T1 and T2 ponderations (bone formation), establishing certainty diagnosis of accessory navicular bone, excluding the suspicion of improperly consolidated navicular fracture/pseudoarthritis. STIR and PD fat sat sequences reveal hypersignal alterations in the posterior tibial tendon tangent to the accessory bone mentioned above, establishing the diagnosis of impingement syndrome with subsequent tendinopathy alterations (Figure 9, Figure 10 and Figure 11).

Excluding the improperly healed fracture of the navicular, the patient was treated as a contusion with functional rest, NSAIDs, analgesics, and physical therapy, which allowed the player to recover early and resume his competitive activity after 1 week.

## 4. Discussion

In this case series of elite handball players, MRI proved indispensable for the accurate diagnosis of high-impact musculoskeletal injuries that were either mischaracterized or undetected by initial clinical assessment and radiography. Across four distinct injury patterns—including vastus lateralis tears, proximal hamstring avulsions, adductor complex strains, and accessory navicular impingement—MRI not only enabled precise anatomical localization and severity grading but also guided the implementation of tailored conservative treatment protocols. These individualized approaches, comprising rest, NSAIDs, physiotherapy, and progressive kinesitherapy, led to full recovery and a timely return to play in each case. Our findings underscore the pivotal role of advanced imaging in optimizing non-operative management strategies for handball-related trauma and highlight the potential to reduce misdiagnosis and rehabilitative delays in high-performance sports.

Muscle tears are defined as a type of shear strain involving both myofibrils and the associated endomysial sheaths. They are classified as grades I, II, and III, according to the number of disrupted fibers. Grade I strains have few torn myofibers, with associated edema minor discomfort, and minimal loss of strength or range of motion. Grade II strains are characterized by clearly reduced strength due to the high number of torn myofibers, whereas grade III strains represent a complete tear of the muscle and myofascial sheaths, resulting in a complete loss of contractile function, often accompanied by a palpable injury [13,14].

Muscle damage can result in substantial hematomas due to the rupture of intramuscular vessels. Heamatomas are considered intramuscular if the epimysium contains the hematoma or intramuscular if the muscle fascia becomes discontinuous. Once the muscular fascia is torn, hematomas tend to fuse inside the adjacent anatomical regions.

Intramuscular hematomas are considered much more severe because the intact fascia leads to increased pressure, which compresses the capillary system as bleeding progresses, generating compartment syndrome with hypoxia and secondary muscle necrosis. Consequently, the prognosis for intramuscular hematomas is much more severe than that for intermuscular hematomas, and experts believe that they should be treated with extreme caution to avoid other complications such as traumatic ossifying myositis [13,14,15].

The quadriceps muscle contains four muscle groups: vastus lateralis (originating from the lateral part of the intertrochanteric line, the margin of the greater trochanter, the lateral margin of the gluteal tuberosity, the lateral lip of the linea aspera), vastus medialis (originating from the medial part of the intertrochanteric line, pectineal line, medial lip of the linea aspera), vastus intermedius (originating from the proximal part of the anterolateral femoral diaphysis), and rectus femoris (with its two-headed tendon, the direct tendon inserted on the anterosuperior iliac spine, and the reflected tendon, inserted inferior and superior on the rim of the acetabulum). The four muscles converge in the occipital tendon, which contains the patella that forms the patellar tendon and is inserted into the tibial tuberosity. The four quadriceps muscle groups function as hip flexors and knee extensors [16]. Eckard et al. reported the incidence of quadriceps muscle damage among college national association players, noting it was less common than hamstring injuries but three times more frequent among female players. During pre-season training sessions, non-contact injuries accounted for 63%, overexertion injuries made up 22%, and the relapse rate was approximately 7.2% in women’s basketball and men’s football [17].

Isolated damage of the vastus lateralis has been reported in the literature as being more common in male players, and in cases of muscle avulsion, reinsertion surgery is recommended. It is relevant that female players seem to have different degrees of muscle damage risk compared to male players, due to anatomical and biomechanical differences [18].

Souza et al., Voight et al. and Cowan et al. suggested, based on electromyography studies, that the altered contractile models of the quadriceps can perturb the dynamic of the patellofemoral joint. LaBore and Weiss suggested that damage to the vastus lateralis impairs the dynamics of the patellofemoral joint. Conservative treatment includes kinetotherapy; however, prophylaxis is the most important treatment, mainly for professional sportspeople. One of the protocols used was the one published by Daneshjoo et al., who recommended both the FIFA +11 and the prevention programs of the HarmoKnee training exercises that combine strength, neuromuscular control, balance, and correct kinematic models without resorting to special equipment, focusing mainly on the isometric strength of the quadriceps [19,20,21,22].

Special attention should be paid to muscle damage acquired during professional sports and an approach should be developed based on the prophylaxis and management of these injuries, including gender-specific training programs, biomechanical assessment, and personalized recuperation strategies [23].

Muscle injuries are common among both genders, yet physiological and biomechanical differences call for gender-specific training programs. In elite athletes, the ACL is frequently injured, with females being significantly more susceptible up to eight times more likely than males [24]. Male athletes, on the other hand, tend to experience more hamstring injuries due to their increased muscle mass during high-intensity sports [25].

Gender-specific training programs should focus on:

- Female athletes: Emphasize knee stabilization exercises, neuromuscular control drills, and balance training to reduce valgus forces. Plyometric exercises can improve knee stability. Training intensity should consider menstrual cycle-related hormonal factors.
- Male athletes: Focus on hamstring strengthening, particularly eccentric exercises like Nordic hamstring curls, alongside agility drills to improve muscular endurance and flexibility.

Regular biomechanical assessments should be conducted to identify movement patterns that may lead to injury, including gait analysis and functional movement screening during high-stress activities.

The ischial calf muscles contribute to hip extension, pelvic stabilization, and knee flexion [26]. In sportspeople, damage to the ischial-calf muscles is the most common type of injury, and its incidence ranges between 3 and 4.1/1000 h of competition. Injuries may vary from muscle elongation to complete tendon tears and are most common at the musculotendinous junction. Among all ischial-calf damage cases, proximal avulsion occurred in 12%, and complete avulsion occurred in 9%. These injuries are treated conservatively using functional recuperation if the retraction is <2 cm or if a single tendon is involved. However, surgery is recommended when the damage involves two or more tendons and retraction is greater than 2 cm [27,28].

Injuries to the proximal part of the ischial calf muscles, which are more common in teenagers and less frequent in adults, can lead to avulsion fractures of the ischial tuberosity. While these injuries are typically managed conservatively, similar to adults with purely tendinous damage, surgery is preferred when there are tears with significant secondary retraction. In acute injuries, surgical management usually consists of open reduction and internal fixation (ORIF) of fractures. In certain cases, these injuries can be mistakenly diagnosed, leading to delayed care and, subsequently, to a chronic lack of consolidation [29].

In the case of chronic unconsolidated lesions, conservative treatment can be initiated, including nonsteroidal anti-inflammatory drugs, physical therapy, and progressive resumption of activity. If this approach fails, then surgery should be considered [29].

Avulsion fractures of the ischial tuberosity are rare injuries that usually occur in players with immature skeletons as a consequence of the eccentric contraction of the ischial-calf muscles caused by sudden and forced flexion of the hip while the knee is extended. Untreated fractures with displacement lead to the development of pseudoarthritis and fibrosis of the proximal ischial calf muscles, resulting in chronic pain, a reduced range of active and passive movements, and altered locomotor biomechanics. The number of reported cases of surgical fixation for these fractures is low. Bone plate fixation offers a stable result but usually requires an extensive Kocher–Langenbeck approach, with possible subsequent damage to the femoral head and blood vessels of the proximal femoral epiphysis. Biodegradable suture anchors can also be used, but failed cases have been reported because of weakening of the suture. Cannulated screws are an alternative with a higher success rate in acute injuries where the displacement of the avulsion fragment is less than 15 mm. However, the results of delayed surgery were less effective than those of immediate posttraumatic fixation [30].

Ferlic et al. described the successful use of three 4 mm cannulated screws in fractures with ischial tuberosity displacement, with immediate post-trauma surgery using a transversal incision, with the patient lying on their side. They reported a case of ischial tuberosity avulsion in a 14-year-old patient who underwent surgery 12 months after the initial injury. Postoperatively, the patient continued to feel pain while sitting, and was never able to resume his sporting activity. Patients with a displacement of <15 mm were treated conservatively, with excellent results [30]. Kaneyama et al. described the use of two 4 mm cannulated screws using a subgluteal approach to successfully treat an acute ischial tuberosity fracture in a 16-year-old patient at a 3-hour interval post-injury. The sciatic nerve was not exposed directly. The recovery time necessary to fully resume sporting activity was 4 months. An alternative approach to avoid metal insertion involves the use of biodegradable suture anchors. Biedert et al. described this technique in three players with ischial tuberosity avulsion fractures during training. However, of the three players, one failed because of suture deterioration [31].

Most authors agree that displacements greater than 15 mm require surgical treatment for ischial tuberosity avulsion fractures, with possible complications entailed by conservative treatment in these patients, including pseudoarthritis and chronic pain secondary to leg shortening and fibrosis at the origin of the ischial calf muscles, with the impossibility of sitting down for longer periods of time. However, the type of surgery best suited for these cases remains a matter of debate [30,32].

The adductor brevis muscle is part of the adductor muscle group, which also includes the pectineus, adductor magnus, gracilis, and adductor longus. The pectineus, adductor brevis, and adductor longus are collectively known as the “short adductors,” connecting the pelvis to the femur. In contrast, the gracilis and adductor magnus, referred to as the “long adductors,” extend from the pelvis to the knee. The main function of these muscles is to bring the pelvic limbs closer together. They also act while sprinting, twisting, or hitting objects.

The adductor brevis originates on the anterior surface of the inferior pubic branch, which is inferior to the origin of the adductor longus. It was inserted into the pectineal line to the superior part of the medial lip of the linea aspera. It is innervated by the branches of the obturator nerve. The obturator and medial femoral circumflex arteries ensured the necessary blood supply to the muscle. Its function is to adduct and flex the thigh and to contribute to lateral rotation [33].

The adductor magnus, pectineus, adductor longus, and adductor brevis muscles can perform both interior and exterior rotations of the thigh. Their rotation function depends on the specific flexion–extension and abduction–adduction positions of the hip joint. Flexion and abduction intensify the lateral rotation function of these muscles, while extension and adduction intensify the medial rotation function.

Clinically, the muscle strain of the adductor group of muscles was as follows: grade 1, slight discomfort, slight sensitivity at a certain point, no swelling; grade 2: greater pain, swelling, tenderness to palpation, reduced movement amplitude, and impaired running; grade 3: very painful, increased swelling, and total loss of running or even walking capability [34].

The commonest causes of pain in the inguinal area are described as being connected to the adductors, the iliopsoas, and inguinal muscles. However, external rotator muscle injuries can also cause pain in the inguinal area and should be considered. Several mechanisms of the damage have been described, but none are very specific, and the injuries described more often involve movements made by the player: swift and unstable changes in speed and load associated with flexion and internal rotation. Despite the various mechanisms described, this damage is believed to develop because of attempts to stabilize the hip during a stressing activity that involves a combination of forces that act on the coxofemoral joint [33,34].

In most cases, players with injuries of the external obturator complain of pain in the anterior part of the hip and rarely of pain in the gluteal region, which could result from any other pathology more common than external obturator damage. This type of injury is often misdiagnosed as injury of the adductor muscles due to the location of the pain that can be connected to the joint innervation of these muscles by branches of the obturator. The most suggestive clinical sign is considered acute or subacute pain in the anterior hip, triggered by instability in highly demanding sports, associated with pain during passive rotation of the hip or against hip resistance at 90^°^ flexion. Another important characteristic is the absence of pain on palpation, because the external obturator is located deep. Despite these characteristics, clinical diagnosis remains a challenge, and ultrasound seldom has sufficient resolution to provide a correct diagnosis. Consequently, MRI is the gold standard for properly assessing the location and severity of the damage. The clinical signs and physical examination findings are non-specific; therefore, diagnosis is ultimately a diagnosis that excludes the most common causes. It requires a well-documented presumptive clinical diagnosis as well as MRI confirmation [35,36].

There is currently no established consensus on rehabilitation protocols for muscle damage at this level. Rehabilitation programs usually last for 2–3 weeks, starting with relative rest and pain control and progressing to range of motion (ROM) and muscle hypertonicity recuperation from static to dynamic exercises, according to the patient’s tolerance. Subsequently, training is resumed with sport-specific exercises until the player is ready to (return to play). Strengthening muscle tone and aerobic training are also important during this process. A structured rehabilitation approach used in these cases is summarized in Table 1 [37].

The prognosis for injuries of the adductor group corroborated with the damage to the external obturator is generally good. The average return-to-play (RTP) duration typically ranges from 18.2 ± 3 days to 11.5 ± 8.8 days, although this may vary. This clinical case confirmed the player’s RTP within 23 days [36,38].

The objective was to achieve strength comparable to that of the uninjured muscles on both sides. The focus shifted to neuromuscular control and advanced strengthening exercises. More dynamic and plyometric exercises were introduced to prepare the players to fully resume their activities.

Once the strength and range of movement have been completely restored and pain is minimal or absent, players can gradually resume their sporting activity. The comeback was closely monitored to ensure that there were no signs of relapse [37].

The accessory navicular is a common variant of the anatomy of the foot, characterized by supplementary ossification or bone prominence at the level of the navicular or tarsal scaphoid. The prevalence of the accessory navicular in children is estimated to be approximately 10–12%. The development of symptoms secondary to the presence of an accessory navicular was reported in 0.1% (1 in 1000) of the adult patients. When symptomatic, patients usually complain of a sensitive and erythematous bony prominence on the medial part of the foot, with exacerbated pain when walking, playing sports, wearing shoes, eversion, and plantar flexion. The diagnosis of an accessory navicular is typically confirmed by the presence of pain in the medial aspect of the foot, as observed on radiographic imaging, which reveals additional ossification of the navicular bone. Current guidebooks recommend an initial conservative treatment, including observation, anti-inflammatory drugs, orthosis, and walking boots; if symptoms persist despite conservative treatment, surgical removal or debriding is recommended [39,40].

So far, it remains unclear why the symptoms of the accessory navicular manifest only in some patients. Previous authors suggested as possible causes of pain the existence of concurrent posterior tibial tendonitis, the inflammation caused by the pressure on the bony prominence, lax ligaments, synchondrosis trauma, and the alteration of the middle foot biomechanics. The condition of flatfoot, also known as pes planus, has been linked to a symptomatic accessory navicular. Some researchers propose that this foot type may exert increased stress on the accessory navicular during load-bearing activities, although this hypothesis remains a subject of debate.

Patients seldom complain of pain before adolescence, and most report symptoms in their second decade of life. Studies that examined the association between skeletal maturity and accessory navicular development demonstrated that female patients become symptomatic much earlier than male patients. However, no study has definitively proved the association between the development of the accessory navicular and the onset of symptoms [40].

The degree of skeletal maturity was directly correlated with the type of accessory navicular, indicating that the type of accessory navicular changes with skeletal development. Type II accessory navicular is histologically joined synchondrosis to the fibrocartilage. Type III is similar in shape and size, but the connection is made of bone and fused to the navicular. This connection may result from the development of type II fibrocartilaginous synchondroses. Longitudinal bone fusion was previously described by Knapik et al., who reported a ratio of natural fusion of 42% (*n* = 8 of 19), and Nakayama et al. described a fusion ratio of 10% to 14% for the type II accessory navicular. Our data support these figures, indicating a decreasing tendency of the type II accessory navicular and an increase of the type III accessory navicular as skeletal maturity progresses. Extensive imaging is required to understand the pattern and natural development of accessory navicular subtypes [41,42].

Surgery remains the common treatment for accessory navicular, as 46% of the patients included in the study underwent at least one surgery (*n* = 33/71). This percentage is lower than that reported by Jegal et al., where surgical treatment was performed in 89% of the patients (*n* = 57/64), and that reported by Grogan et al., in which surgery was performed in 77% of the patients (*n* = 17/22). However, Grogan et al. mentioned using conservative treatment in only 41% of patients with accessory navicular fractures (*n* = 16/39). However, Jegal et al. recommended at least 6 months of conservative management, but 37% (*n* = 29/79) of the patients were sports players who probably opted for surgery to speed up the improvement of the symptoms and to resume their sporting activity [39,41].

Posterior ankle impingement syndrome (PAIS) is a common injury in players with repeated plantar flexion. Although the two-portal posterior approach for the posterior foot has gained popularity, it remains a topic of considerable debate due to its technical complexity, relatively steep learning curve, and the challenges associated with performing simultaneous arthroscopy of the anterior ankle. The aim of a possible reassessment of the data presented in the literature is to offer comprehensive information about PAIS and to describe a systematic approach in four stages of two-portal posterior arthroscopy. Etiology, clinical presentation, and diagnostic strategies have been introduced from the beginning, followed by various conservative and surgical management options. The study continues with a detailed description of the arthroscopy of the posterior foot. This technique allows for a systematic examination of the anatomical structures and treatment of bone/soft tissue damage in four regions of interest of the posterior foot (superolateral, superomedial, inferomedial, and inferolateral). The review focuses on biological accessory therapy and postoperative rehabilitation and ends with a discussion about the latest clinical results of arthroscopy of the posterior foot in PAIS. Although clinical evidence suggests high short- and medium-term success rates after arthroscopy of the posterior foot, its use may be limited owing to the required technical abilities, which can be improved by the systematic approach of two-portal posterior arthroscopy [43,44,45].

The findings of this study provide valuable insights into injury prevention and rehabilitation strategies for professional handball players. Coaches and sports scientists can apply the diagnostic and treatment approaches outlined in this research to enhance athlete performance and longevity.

Based on the frequent knee and muscle injuries identified, it is recommended that handball training programs integrate targeted strength and stability exercises. For instance, incorporating proprioceptive training—such as balance exercises using single-leg stances or unstable surfaces like BOSU balls—can enhance joint stability. Furthermore, neuromuscular control drills that involve controlled jump landings and lateral cutting exercises are crucial for improving dynamic knee stability. Emphasizing the strengthening of key muscle groups through eccentric hamstring exercises, quadriceps conditioning, and hip abductor training can help prevent muscle strains and maintain balanced joint support.

The conservative treatment methods discussed, particularly the use of MRI for accurate diagnosis and monitoring, underscore the importance of individualized rehabilitation plans. Coaches and rehabilitation experts should incorporate regular MRI diagnostics to assess injury severity accurately and track healing progress, especially when initial clinical evaluations are inconclusive. A structured, stage-based rehabilitation protocol—comprising acute, subacute, remodeling, and functional phases—ensures that athlete readiness for progressive load-bearing exercises is continuously assessed.

By integrating these diagnostic tools and therapeutic strategies into daily practice, coaches can reduce the risk of recurring injuries and ensure a safer, more effective return to sport for handball players.

## 5. Limitation of the Study

However, several limitations must be acknowledged. Our prospective design and small sample size (*n* = 4) restrict the generalizability of these findings, as the selected cases represent specific injury types common in handball and may not capture the full variability in injury severity and outcomes across a larger population. Furthermore, the cases were chosen based on availability during the research period rather than through a structured sampling method, so generalizing these findings to the broader handball community or other sports should be approached with caution. Additionally, variability in treatment outcomes—potentially influenced by factors such as age, physical condition, and injury history—could not be fully controlled. A larger cohort study is necessary to validate these preliminary findings and provide a more comprehensive understanding of injury management in handball players. Similar small-series investigations in elite athletes—such as Carrozzo et al.’s three-athlete case report on non-surgical adductor longus tears and Biedert et al.’s three-case series of ischial tuberosity avulsions—face comparable challenges in statistical power and risk of selection bias [31,37]. Purposive case selection, while illustrative, may further introduce bias. Prospective cohort studies with larger samples and control groups are needed to validate MRI-guided conservative management protocols in professional handball injuries.

## 6. Future Directions

Building on the insights gained from our four-case series, future research should pursue several avenues to strengthen the evidence base for MRI-guided conservative management in elite handball:

First, prospective, multicentre cohort studies enrolling larger numbers of athletes will be essential to overcome the limitations of small sample sizes and single-center bias. Such studies should harmonize inclusion criteria, imaging protocols, and rehabilitation regimens across sites to facilitate pooled analysis.

Second, long-term follow-up extending beyond the initial six-week recovery period is needed to capture delayed complications, recurrence rates, and sustained functional outcomes. Regular clinical, functional (e.g., LEFS, isokinetic testing), and imaging (e.g., MRI edema quantification) assessments at 3, 6, and 12 months post-injury will provide a more comprehensive picture of rehabilitation durability.

Third, comparative trials contrasting conservative versus surgical interventions for specific injury types—such as high-grade muscle tears or avulsion fractures with >15 mm retraction—could delineate which lesions benefit most from non-operative care. Incorporating patient-reported outcomes (e.g., sport-specific questionnaires, quality of life scales) will ensure the athlete’s perspective guides treatment decisions.

Fourth, integrating advanced quantitative imaging biomarkers—such as diffusion-weighted MRI for muscle microstructure or T2-mapping for tendon integrity—may refine prognostic models and personalize rehabilitation intensity and duration.

Finally, mechanistic studies examining the interplay of neuromuscular control, biomechanical loading, and tissue healing in handball athletes can inform targeted injury-prevention programs. Collaboration between sports physicians, radiologists, physiotherapists, and performance scientists will be key to translating these future directions into improved athlete care.

## 7. Conclusions

Musculoskeletal injuries are common in high-performance sports like handball, where the physical demands greatly increase the risk of injury. This study underscores the importance of accurate diagnostic methods, particularly MRI, which corrected initial misdiagnoses in most cases. Conservative treatments, such as rest, NSAIDs, physical therapy, and kinesitherapy, proved effective in promoting early recovery and facilitating a timely return to competition. These findings highlight the need for individualized therapeutic approaches to optimize recovery and ensure a safe return to sports participation. Future research should broaden the investigation of injury types beyond those covered here, incorporate longitudinal studies to assess long-term treatment efficacy and recurrence, and include randomized controlled trials or multicenter studies with larger samples. Additionally, exploring how these diagnostic and rehabilitation strategies can be applied to other high-impact sports will provide valuable insights into sports medicine and enhance athlete care across various disciplines.

## Figures and Tables

**Table 1:** Rehabilitation protocol for handball-related musculoskeletal injuries [37].

Rehabilitation Stage	Objective	Example Exercises	Description
Stage 1: Acute Phase (Days 1–4)	Pain control, swelling reduction	-Cryotherapy -Rest and NSAIDs	Focus on reducing inflammation and preventing further damage. Ice packs applied for 15–20 min intervals.
Stage 2: Subacute Phase (Weeks 1–4)	Regain flexibility, isometric strength	-Passive ROM exercises -Isometric quadriceps contractions -Gentle stretching	Begin gentle movement without putting too much stress on injured muscles. Gradual introduction of pain-free stretching.
Stage 3: Remodeling Phase (Weeks 3–5)	Strengthen muscles, improve range of motion	-Concentric and eccentric exercises -Squats and lunges (modified, if needed)	Focus on rebuilding muscle strength with gradual progression in intensity. Maintain a balance between flexibility and strength.
Stage 4: Functional Phase (Weeks 6–8)	Full muscle strength, return to play	-Plyometrics (box jumps, lateral jumps) -Sport-specific drills	Include sport-specific movements to ensure that the athlete is ready to return to competitive activity. Monitor for signs of relapse during this phase.

## Data Availability

The original contributions presented in this study are included in the article. Further inquiries can be directed to the corresponding author(s).
